# MSM enhances LPS-induced inflammatory response after exercise

**DOI:** 10.1186/1550-2783-12-S1-P48

**Published:** 2015-09-21

**Authors:** Simone Godwin, Richard J Bloomer, Marie van der Merwe, Rod Benjamin

**Affiliations:** 1 Department of Health and Sport Sciences, University of Memphis, Memphis, TN, USA; 2 Bergstrom Nutrition, Vancouver, WA, USA

## Background

Methylsulfonylmethane (MSM) has been reported to positively influence markers of inflammation and exercise recovery, including decreasing muscle soreness and fatigue. Acute exercise induces tissue damage that results in sterile inflammation that is propagated by secreted mediators such as IL-6 and TNF-α. Regulation of the inflammatory response is critical as chronic inflammation is associated with a plethora of diseases. In addition to the exercise recovery, MSM has also been reported to reduce inflammation associated with osteoarthritis and allergy. Based on these data we designed a pilot study to determine the effect of MSM on Lipopolysaccharide (LPS) - induced inflammatory mediators after a single bout of acute eccentric exercise.

## Methods

Blood was collected from five recreationally active, healthy men after 28 days of supplementation with MSM (OptiMSM^®^; Bergstrom Nutrition, Vancouver, WA) or placebo (rice flour) indicated by "Base". Subjects #19 and #20 received placebo, while #36, #39 and #40 received 3 g of MSM per day. A single bout of acute exercise (10 sets of 10 repetitions of eccentric knee extensions) was performed and additional blood samples were collected immediately (0 h) and 24 h, 48 h and 72 h post exercise. 250 µl of whole blood was plated in a 96-well U bottom plate containing 50 µl of tissue culture media (RPMI1640, antibiotics, 10% FBS) with or without LPS (final concentrations = 0.2 ug/ml). The samples were then incubated at 37°C for 24 h and plasma collected by centrifugation and stored at -80°C until analysis. Plasma cytokine concentrations were determined using a MILLIPLEX MAP human custom cytokine magnetic bead panel that included analytes for IL-1β, IL-6, IL-10, IL-17a and TNF-α. Analytes were quantified using a MAGPIX^® ^and xPONENT software.

## Results

The supplementation of MSM blunted the increase in the systemic levels of inflammatory cytokines (IL-6 and IL-1β) immediately after exercise. *Ex vivo *incubation of blood from various time points with LPS, caused a dramatic increase in inflammatory cytokine secretion (IL-6, IL-1β and TNF-α) only after exercise for samples that was exposed to MSM. This response is specific to the stimulation with LPS as secretion of LPS-non responsive proteins is not increased, as evident by the stable levels of IL-17a. There is also a 2-3 fold increase in IL-10 production after LPS stimulation for the MSM group despite having lower IL-10 levels before exercise.

## Conclusion

MSM is able to reduce the initial cytokine surge that is induced by acute exercise, while allowing for an efficient response to infectious stimuli after a single bout of acute exercise.

